# Conjugated photothermal materials and structure design for solar steam generation

**DOI:** 10.3762/bjnano.14.36

**Published:** 2023-04-04

**Authors:** Chia-Yang Lin, Tsuyoshi Michinobu

**Affiliations:** 1 Department of Materials Science and Engineering, Tokyo Institute of Technology, 2-12-1 Ookayama, Meguro-ku, Tokyo 152-8552, Japan

**Keywords:** absorption, conjugated molecules, energy transfer, photothermal materials, solar steam generation

## Abstract

With the development of solar steam generation (SSG) for clean water production, conjugated photothermal materials (PTMs) have attracted significant interest because of their advantages over metallic and inorganic PTMs in terms of high light absorption, designable molecular structures, flexible morphology, and solution processability. We review here the recent progress in solar steam generation devices based on conjugated organic materials. Conjugated organic materials are processed into fibers, membranes, and porous structures. Therefore, nanostructure design based on the concept of nanoarchitectonics is crucial to achieve high SSG efficiency. We discuss the considerations for designing SSG absorbers and describe commonly used conjugated organic materials and structural designs.

## Review

### Introduction

With the rapid development of the world economy, global water shortages are occurring. Current technologies for dealing with the water shortage problem either exacerbate energy problems or sacrifice the environment. Solar-driven steam generation technology, in contrast, is a solar-powered technology that meets the global trend for clean, sustainable, and green technology.

**Figure 1:**
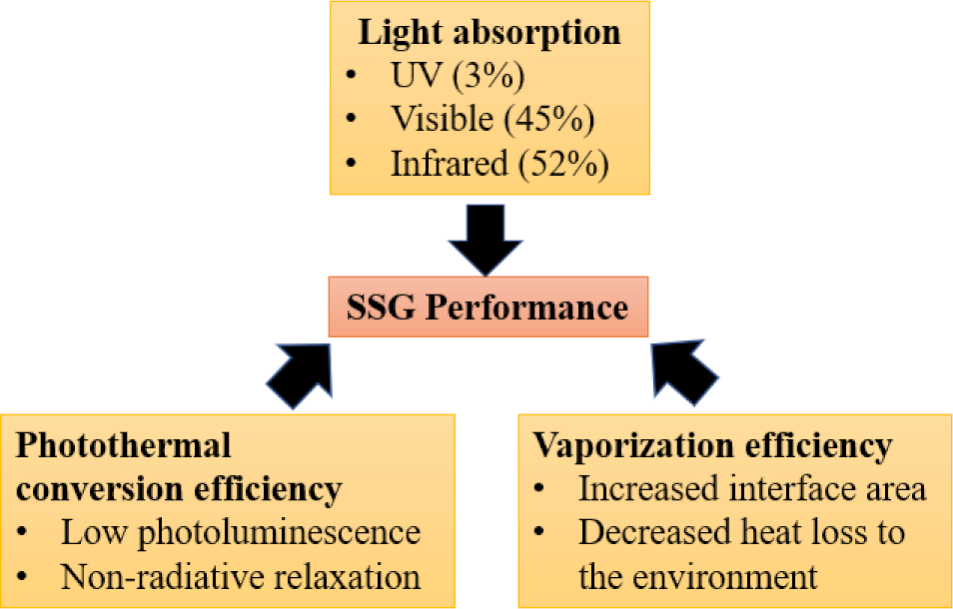
Design concept for high-performance SSG devices.

To obtain a high efficiency in solar steam generation (SSG), three factors must be considered, namely solar light absorption, photothermal conversion efficiency, and vaporization efficiency (Figure 1). In addition, the cost of the materials must be taken into consideration as large quantities at low cost are required. Photothermal materials (PTMs) applied to SSG include metallic materials, semiconductors, carbon-based materials, and conjugated organic materials [1–4]. Compared to metallic and inorganic PTMs, π-conjugated organic PTMs have advantages, such as a greater light absorption, easier synthesis, and tunability of molecular structures [5–6]. In fact, there is a long development history of conjugated molecules and polymers, and a clear correlation between their chemical structure and physical properties has been established [7–17]. For example, the absorption spectra can be predicted by density functional theory (DFT) calculations of the conjugated structures. This is an advantage over other carbon materials, even if they are of the same chemical composition [18–22].

We review the recent progress in the material development of conjugated solar absorbers based on (1) various molecular designs of small molecules and polymers, (2) absorber structures from a thermal management perspective, and (3) applications to desalination, sterilization, wastewater treatment, and power generation. As we focus on the SSG application in this review, the other applications will be briefly discussed in the last part.

### Solar steam generation absorbers

Numerous studies have been conducted of various materials and structures of solar energy absorbers for SSG. To achieve a high conversion efficiency in solar steam generation, three key factors must be considered. The first one is sunlight absorption. PTMs must be able to efficiently absorb light in order to utilize the irradiation energy. The second one is the photothermal conversion efficiency. Absorbed light needs to be converted to heat energy rather than other forms of energy loss. The last one is the efficiency of the steam generation from heat. The generated thermal energy must be used for water evaporation and heat loss to the environment needs to be avoided.

#### Sunlight absorption

**Figure 2:**
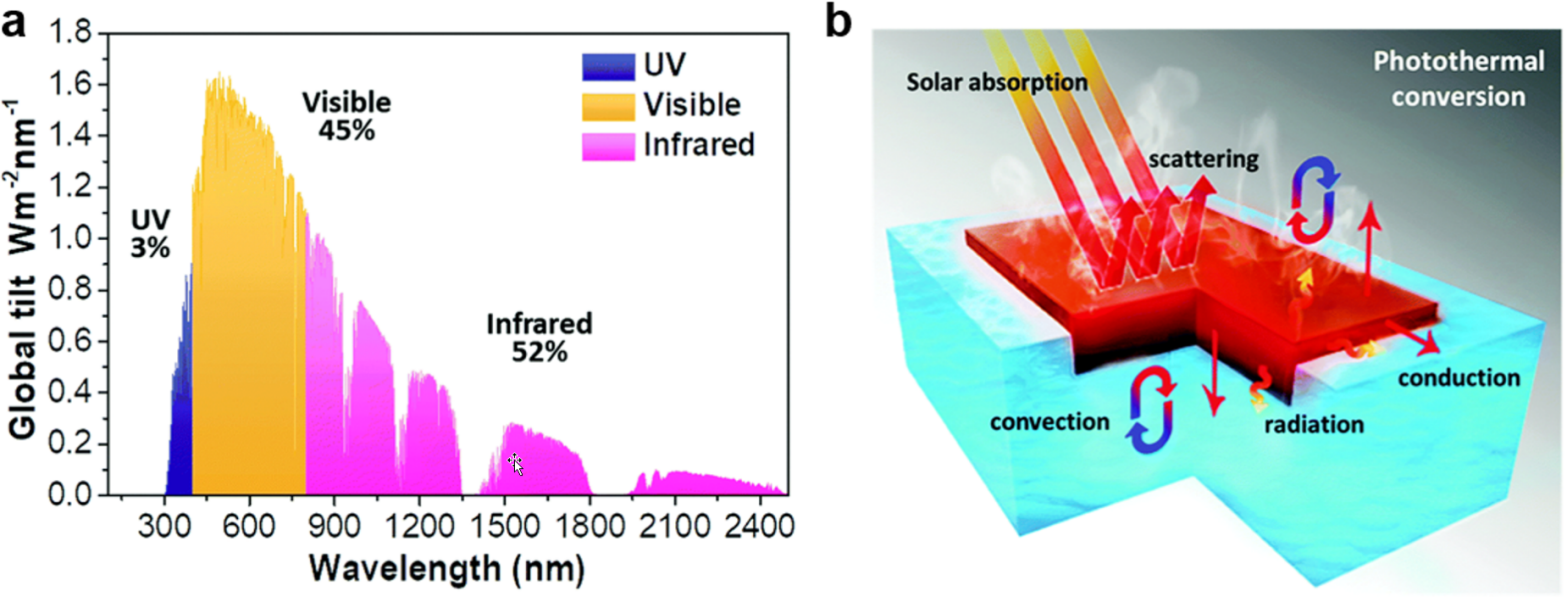
(a) Solar spectral irradiance (AM1.5) with the fractions of each wavelength region. (b) Schematic illustration of photothermal conversion processes for solar steam generation. Figure 2 was adapted with permission of The Royal Society of Chemistry from [23] (“Solar absorber material and system designs for photothermal water vaporization towards clean water and energy production” by M. Gao et al., Energy Environ. Sci., vol. 12, issue 3, © 2018); permission conveyed through Copyright Clearance Center, Inc. This content is not subject to CC BY 4.0.

The wavelength range and intensity of the solar spectrum are important factors for harnessing solar energy. As this review focuses on solar energy applications for water evaporation, the light absorbing properties of materials are intended to achieve the highest conversion of sunlight radiation at sea level. The AM1.5 standard represents the average annual solar radiation at the temperate latitudes (Figure 2a). Therefore, the AM1.5 standard is used in solar cell engineering with a fraction of 3% in the ultraviolet (UV) region (300–400 nm), of 45% in the visible region (400–700 nm), and of 52% in the near-infrared (NIR) and infrared region (700–2500 nm). This suggests that PTMs need to have a high absorption at wavelengths from 300 to 2500 nm and reflectance, transmission, and radiative relaxation must not be significant in order to maximize the energy absorbed from the sun and convert it into thermal energy (Figure 2b).

#### Photothermal conversion efficiency

Solar energy can be converted into various forms of energy such as electricity, chemical energy, and heat through photovoltaic, photochemical, and photothermal processes, respectively. Even though electricity can be converted into thermal energy, it is less efficient than the direct photothermal process. Photothermal effects are produced by electronic excitation and nonradiative relaxation of excited electrons to the ground state. Depending on the interaction mechanism, photothermal phenomena are classified into three categories, namely plasmonic local heating of metals, nonradiative relaxation of semiconductors, and thermal vibration relaxation of conjugated molecules.

**Figure 3:**
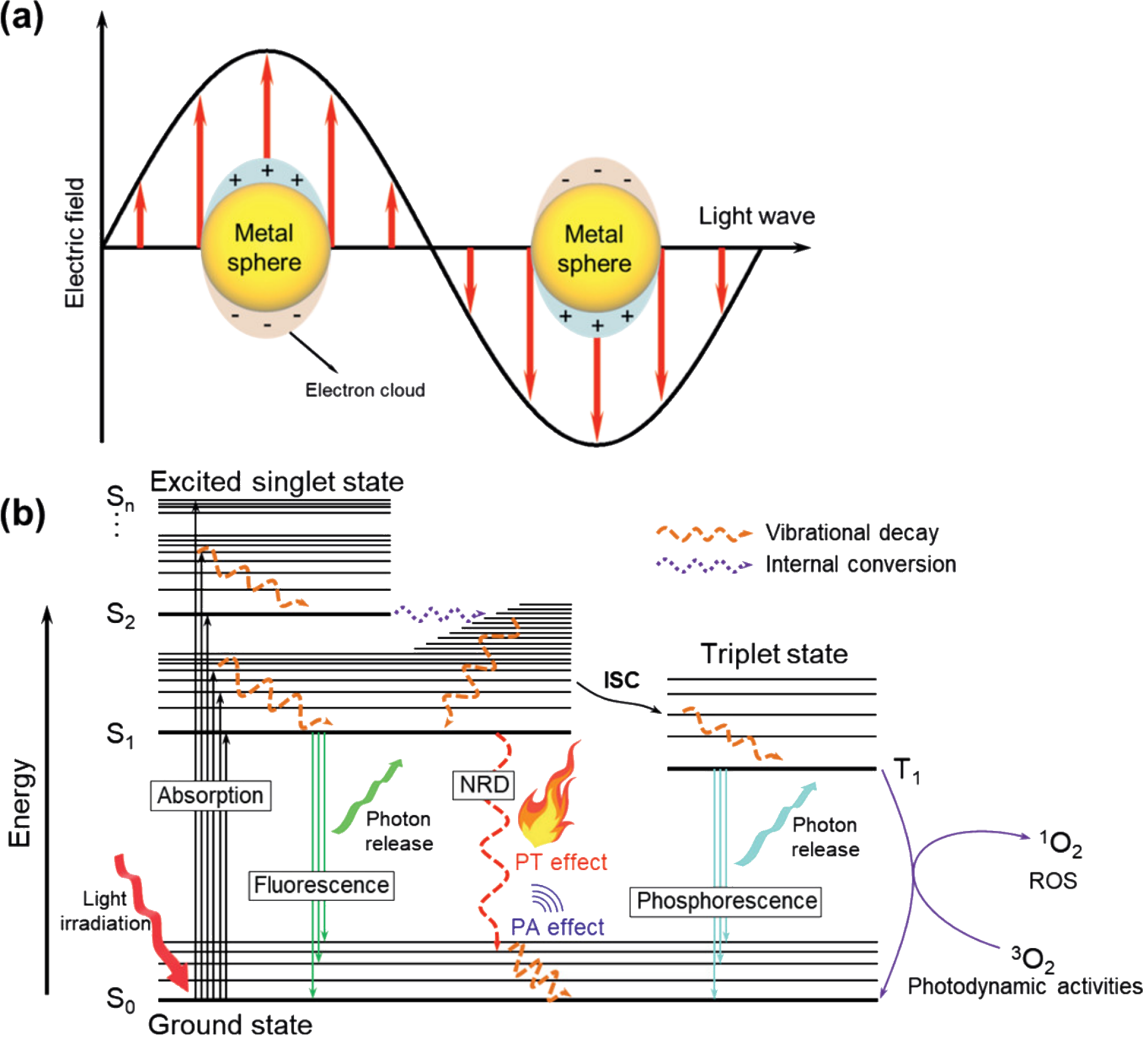
(a) The oscillation of electron clouds in a metal nanoparticle induced by light irradiation. (b) The Jablonski diagram representing the photophysical processes of conjugated molecules including the photothermal effect. Figure 3 was reproduced from [5] (© 2021 H. J. Kim et al., published by Wiley-VCH GmbH, distributed under the terms of the Creative Commons Attribution NonCommercial 4.0 International License, https://creativecommons.org/licenses/by-nc/4.0). This content is not subject to CC BY 4.0. Part (a) of the source was redrawn from [24] and part (b) was inspired by [25].

The free electrons of metallic nanomaterials absorb light. This is followed by specific oscillations that give the photothermal effect. This effect originates from the surface plasmon resonance (SPR) of electrons. The SPR-based thermal energy is then transferred to lattice phonons (Figure 3a).

In semiconductor materials, optical absorption significantly varies with the wavelength, depending on the bandgap energy. When semiconductor materials are irradiated with light, electron–hole pairs with energies close to the bandgap are produced. The excited electrons eventually return to a lower energy state and either undergo radiative relaxation in the form of photons or nonradiative relaxation in the form of phonons (heat) to release and transfer energy to impurities/defects or dangling bonds on the material surface. When energy is released in the form of phonons, local heating of the lattice is induced, yielding a temperature profile based on the optical absorption and bulk/surface recombination properties. Here, the photothermal effect refers to this temperature distribution caused by the diffusion and recombination of optically excited carriers.

In organic materials, the delocalization of electrons in π-conjugated bonds creates the primary carriers that absorb light and generate thermal energy. This is because the π bonds are usually much weaker than the σ bonds (e.g., C=C π bond energy = 272 kJ·mol^−1^, C–C σ bond energy = 439 kJ·mol^−1^) [5]. After excitation by light above the bandgap, electrons in organic PTMs are activated from the S_0_ state to other states (S_1_, S_2_,…, S*_n_*) (Figure 3b). When electrons return to the S_0_ state by nonradiative processes, energy is released in the form of heat. Therefore, the energy level design of PTMs is very important in order to improve their efficiency.

#### Photothermal evaporation efficiency

To achieve the maximum vaporization efficiency, the heat generated by the solar absorber must be fully utilized to vaporize water. However, under actual conditions, the bulk heating of water consumes heat and causes losses to the environment through conduction, convection, and radiation. Therefore, the design of various absorber structures has been studied to minimize any undesirable heat losses.

The light-to-water vapor energy conversion efficiency of SSG can be calculated using the following equations:




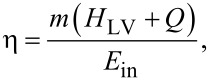







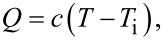







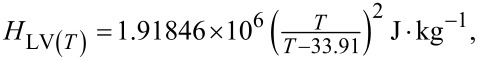




where *m* is the water evaporation rate, *T* is the temperature of evaporation, *T*_i_ is the initial temperature of water, *c* is the specific heat of water (4.2 J·g^−1^·K^−1^), *Q* is the sensible heat of water, *H*_LV_ is the enthalpy of water vaporization, and *E*_in_ is the energy of the incident light.

### Conjugated organic photothermal materials

#### Conjugated organic small molecules

Organic molecular dyes, such as cyanines, croconaines, and diketopyrrolopyrroles (DPPs), have been evaluated for PTMs because of their intense absorption in the NIR range. These materials consist of long conjugated groups that are bridging electron donors or acceptors. The absorption properties of these PTMs can be tuned by changing the donor units that are conjugated with the bridging group. These conjugated small molecules have the advantages of a facile synthesis, abundant derivatives, and tunable properties. However, they have short lifetimes due to low photostability. In order to overcome this issue, rigid conjugated structures are often adapted. For example, it is known that chromophores, such as rylene-based dyes and donor–acceptor conjugated frameworks, can extend the absorption band range and enhance the photostability [26].

**Figure 4:**
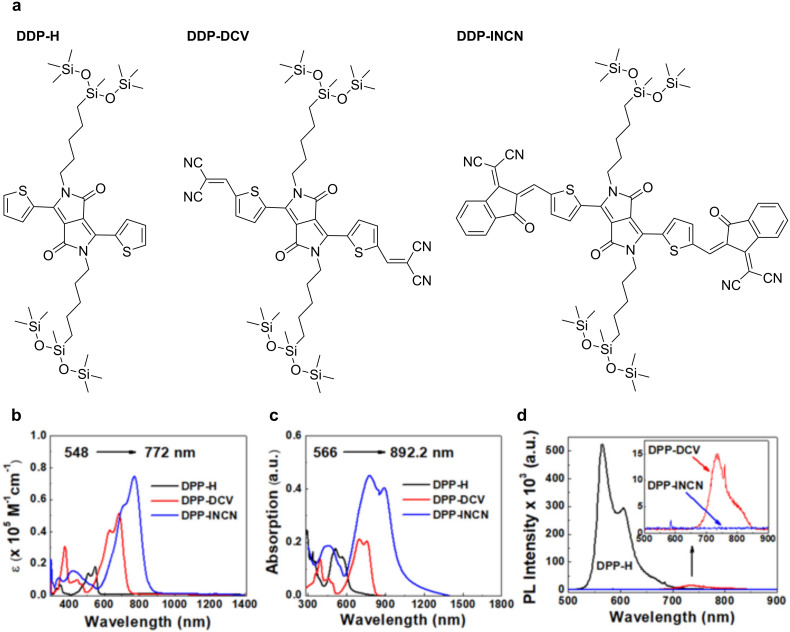
(a) Chemical structures of DPP-H, DPP-DCV, and DPP-INCN and optical absorption spectra of the DPP derivatives (b) in THF solution and (c) for drop-cast films. (d) The photoluminescence (PL) spectra of the DPP derivatives in THF solution. (Figure 4 was adapted with permission from [27], Copyright 2021 American Chemical Society.)

DPP dyes have a strong optical absorption and offer easy alteration of their photophysical properties and hydrophobicity through organic reactions. In general, DPP dyes have high photoluminescence quantum yields, but they have a relatively low photostability, degrading after 150 min under a collimated 300 W Xe lamp light source. It was previously reported that introducing electron-withdrawing (EW) groups, such as malononitrile (DCV) and 2-(3-oxo-indan-1-ylidene)malononitrile (INCN) to the DPP core enhances the stability of DPP dyes [27]. In addition, when a thienyl spacer was introduced between the DPP and EW groups, efficient intramolecular charge transfer (ICT) interactions were generated to yield robust PTM materials suitable for SSG applications. Since DPP-DCV and DPP-INCN exhibited stronger ICT interactions than DPP-H, they showed better photothermal properties (Figure 4a). There results highlight the significance of the chemical structure. DPP-H has solubilizing siloxane chains but not EW end groups. In contrast, DPP-DCV and DPP-INCN have -DCV and -INCN-based EW end groups, respectively. The absorption spectra of the DPP derivatives bathochromically shifted due to the ICT band, and its extinction coefficient increased in the order from DPP-H to DPP-DCV and further to DPP-INCN (Figure 4b and Figure 4c). This suggests that stronger EW end groups and longer conjugation lengths induce more potent ICTs for the DPP derivatives, resulting in smaller bandgaps. In the photoluminescence (PL) spectra of the DPP derivatives, DPP-DCV displayed a much weaker PL than DPP-H, and no PL was observed in DPP-INCN (Figure 4d). This result indicates that DPP-DCV and DPP-INCN have a lower radiative energy loss and the excitation energy is more efficiently converted to heat via nonradiative relaxation.

#### Conjugated organic polymers

**Figure 5:**
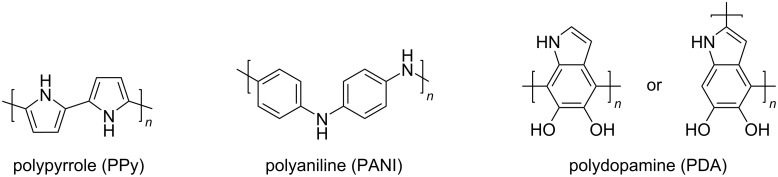
Typical polymeric PTMs used for SSG.

Compared to the small molecular PTMs, polymeric PTMs have the advantage that the π electrons delocalized over the conjugated backbone enhance the carrier transport and provide multifunctional properties by combining their intrinsic optoelectronic properties and macromolecular physical properties. Typical polymeric PTMs used for the SSG include polypyrrole (PPy), polyaniline (PANI), and polydopamine (PDA) (Figure 5).

**Polypyrrole:** Polypyrrole (PPy), a conjugated conducting polymer, has a broad optical absorption spectrum and is an excellent solar thermal material. PPy is a promising candidate because of its inexpensive and simple synthetic process, chemical and thermal stabilities, and insolubility in water and other conventional solvents. Therefore, several studies have applied PPy as a PTM for SSG [28–33].

**Figure 6:**
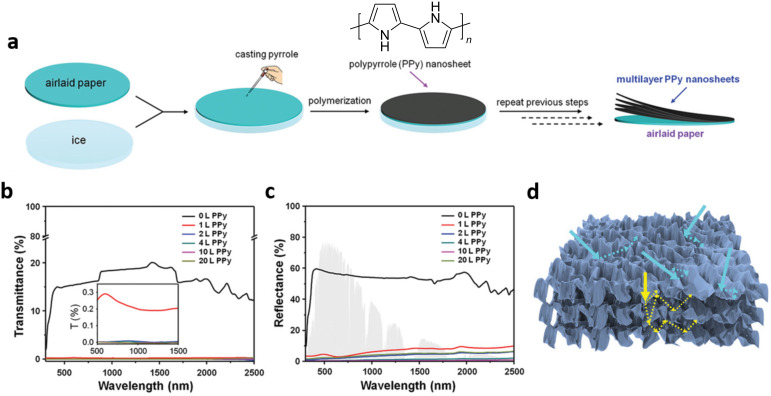
(a) Schematic illustration of the fabrication process of multilayer PPy nanosheets. (b)Transmittance and (c) diffuse reflectance spectra of the air-laid paper substrate coated with different layer numbers of PPy nanosheets. The inset figure in (b) is the enlarged view of the 500–1500 nm wavelength range. The solar spectral irradiance (AM1.5G) is included for comparison in (c). (d) Schematic illustration of light trapping by surface structures formed on the multilayer PPy nanosheets. Figure 6 was adapted from [32], X. Wang et al., “Multilayer Polypyrrole Nanosheets with Self-Organized Surface Structures for Flexible and Efficient Solar–Thermal Energy Conversion”, Adv. Mater., with permission from John Wiley and Sons. Copyright © 2019 WILEY-VCH Verlag GmbH & Co. KGaA, Weinheim. This content is not subject to CC BY 4.0.

In 2019, Wang et al. fabricated multilayered PPy nanosheets with surface structures of wrinkles and ridges, spontaneously formed by sequential polymerization on paper substrates, to enhance their light capturing capability (Figure 6a) [32]. Transmission and diffuse reflectance measurements of the PPy nanosheets confirmed that the multilayer PPy nanosheets on the paper substrate exhibited a distinct broadband absorption in the spectral range of terrestrial solar irradiation (Figure 6b and Figure 6c). Single-layer PPy nanosheets transmit 0.2–0.3% of light, while two or more layers of the PPy nanosheets exhibit a very low transmittance over the entire solar spectrum. It is interesting to note that the light absorption depends on the number of layers, which was caused by the surface structure of the multilayer PPy nanosheets. The high roughness and sharp local curvature of the multilayer PPy nanosheets were retained and incident light was effectively redistributed. In other words, transmitted light is confined in the multilayer PPy nanosheets, and this results in internal light scattering between the layers (Figure 6d). These multilayer nanosheets promote a broadband and wide-angle light absorption across the entire solar spectrum, thus increasing the solar thermal conversion efficiency to 95.33%.

**Polyaniline:** Polyaniline (PANI) has proven to be an efficient water evaporation material because it is inexpensive, easy to synthesize, flexible, chemically stable, light absorbing, and adhesive [34–41]. PANI cross-linked to hydrophilic soft polymers makes the material as tough and flexible as an animal dermis and ensures its long-term applicability for actual solar steam generation [42].

**Figure 7:**
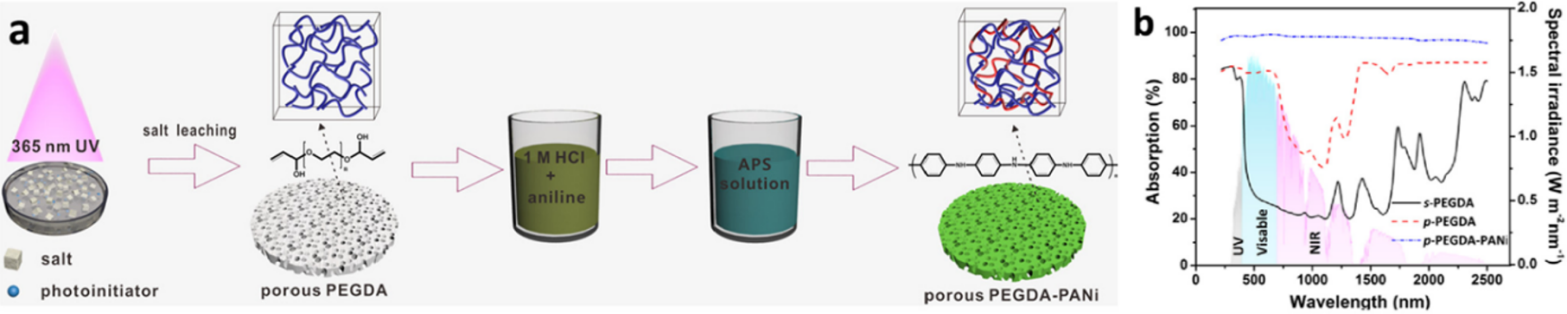
(a) Schematic illustration of the fabrication process and characterization of the photothermal p-PEGDA−PANI hydrogel. (b) Solar spectrum and UV–vis–NIR absorption spectra of the hydrogels. (Figure 7 was adapted with permission from [35], Copyright 2018 American Chemical Society).

In 2018, Yin et al. reported a poly(ethylene glycol) diacrylate (PEGDA) and PANI-based photothermal double-network hydrogel called p-PEGDA-PANI [35]. Porous PEGDA (p-PEGDA) hydrogels were obtained by a facile solvent casting/particle leaching process. Then, the polymeric photothermal material PANI nanowires were cross-linked to the p-PEGDA hydrogels. This manufacturing process is simple, time-saving, and cost-effective (Figure 7a). The strong absorption capability of PANI was verified by its absorption spectrum (Figure 7b). The average absorbance (weighted by the AM1.5G solar spectrum) of the p-PEGDA hydrogel from 200–2500 nm was about 75.5%, whereas that of solid PEGDA is only 32.6%. The higher absorption capacity of p-PEGDA is due to its rough and porous surface structure, which promotes multiple scattering. After crosslinking PANI, the porous hydrogel sample exhibited a broader and stronger absorption (98.5%) than the pure PEGDA sample, especially in the visible and near-infrared regions.

**Polydopamine:** Polydopamine (PDA) has shown great potential in the field of solar-driven desalination due to its broad light absorption, high photothermal conversion efficiency, biocompatibility, and hydrophilicity. The resulting polymer is highly conjugated due to the aromatization of the ethylamine moiety via cyclization and, accordingly, PDA is widely used as a PTM for SSG applications [43–50].

**Figure 8:**
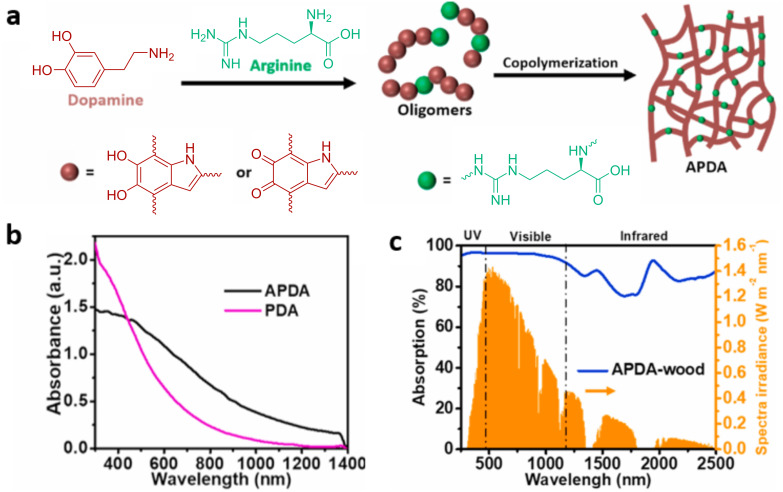
(a) Schematic illustration of the synthetic route to APDA. (b) UV–vis–NIR absorption spectra of APDA and PDA. (c) Absorption spectrum of the APDA–wood composite in the 250–2500 nm range. The solar spectrum is also shown for comparison. Figure 8 was reprinted from [47], Polymer, vol. 217, by Y. Zou; P. Yang; L. Yang; N. Li; G. Duan; X. Liu; Y. Li, “Boosting solar steam generation by photothermal enhanced polydopamine/wood composites“, article no. 123464, Copyright (2021), with permission from Elsevier. This content is not subject to CC BY 4.0.

In 2021, Zou et al. developed a biodegradable and sustainable bilayer composite for highly efficient solar evaporation based on a photothermally enhanced arginine-doped polydopamine (APDA) and raw wood [47]. In this study, APDA coatings with enhanced photothermal effects were prepared by constructing electron donor–acceptor pairs. Density functional theory (DFT) simulations indicated donor–acceptor interactions between arginine and PDA subunits, including the formation of 5,6-dihydroxyindole (DHI) and indole-5,6-quinone (IQ). Dopamine and arginine were copolymerized in an aqueous solution at room temperature to produce APDA. During the polymerization, DHI or IQ moieties were formed by rearrangement of dopamine. The formed DHI and IQ moieties then reacted with each other or the amine groups of arginine. This was followed by further polymerization of DHI, IQ, arginine, and their oligomers, eventually resulting in APDA derivatives (Figure 8a). The formed APDA derivatives displayed a strong and broad absorption in the range of 500–1400 nm (Figure 8b). APDA was then combined with wood. It was coated on the surface of a wood piece and allowed to dry under ambient conditions. This simple process produced the homogeneous formation of the APDA layer on the wood surface. The APDA-coated wood sample efficiently absorbed UV–visible light to more than 96%. In addition, the NIR absorption efficiency was also improved to more than 87% (Figure 8c).

### SSG structure

In order to obtain a high vaporization efficiency, solar energy absorbers with various structures have been fabricated. Different structures have their advantages. Overall, the main purpose is to increase light absorption, increase evaporate interface area, and minimize heat loss to the environment. Janus structures are often applied to SSG absorbers. Half of the surface consists of hydrophilic groups and the other half is hydrophobic groups. Through capillary action, the hydrophilic section sequentially supplies water to the evaporation region, while the hydrophobic segment regulates the surplus water that leads to heat loss in the bulk water [51].

#### Nanofibers

Polymer-based nanofibers can be produced from various processable polymers, and they have wide application possibilities with improved physical properties compared to the pristine polymer solids. Polymer-based nanofibers have characteristic properties, such as a high surface-to-volume ratio, high porosity, and high mass transport. Therefore, they are often applied to SSG absorbers along with other macrostructures such as membranes and foams [29,52–55].

**Figure 9:**
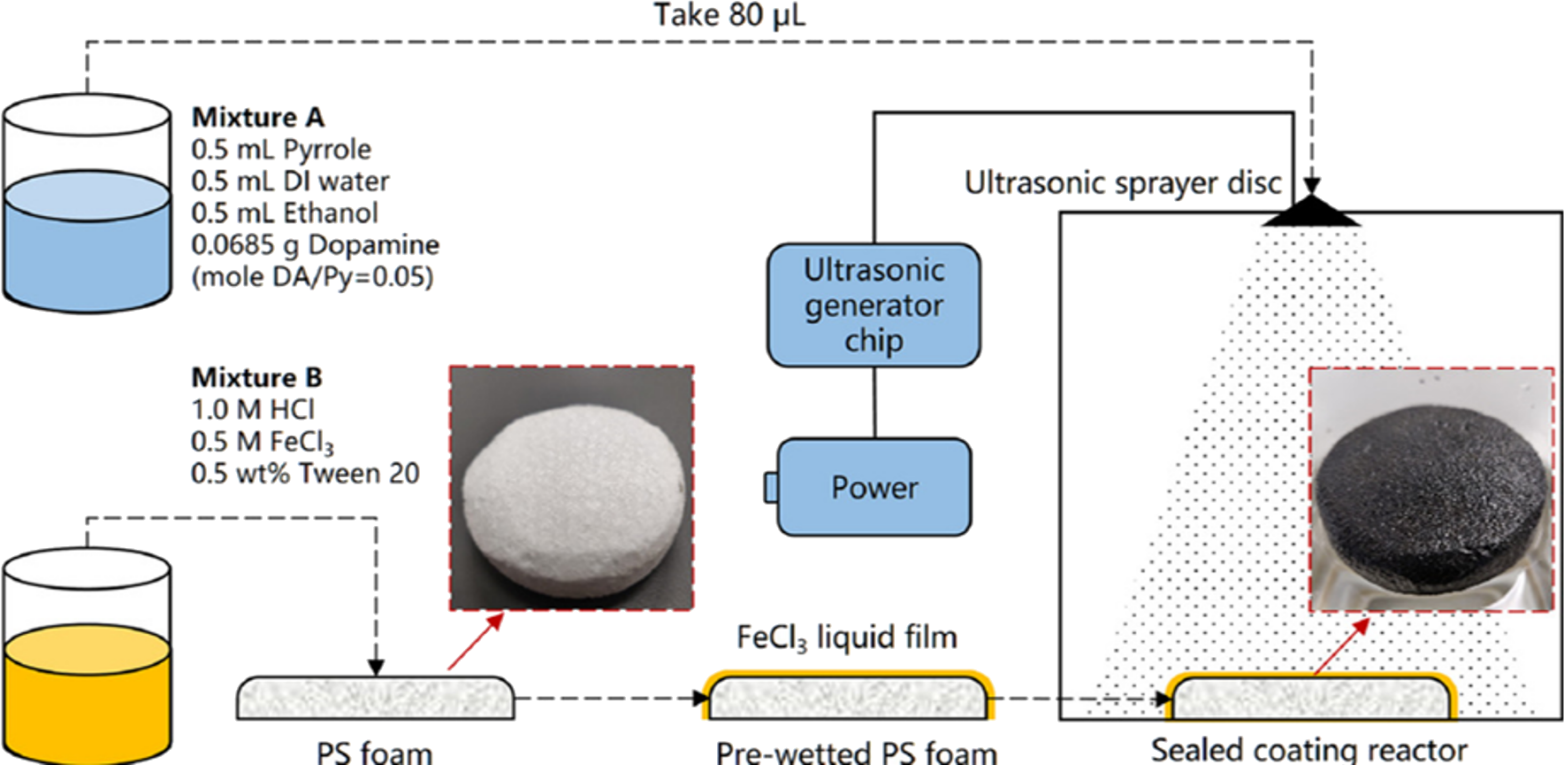
Fabrication process of PPy nanofiber light-trapping coatings by ultrasonic spray coating. (Figure 9 was reproduced with permission from [29], Copyright 2021 American Chemical Society).

One noticeable example is the study of nanofiber-based light-trapping coatings [29]. Ma et al. proposed an ultrasonic spray coating method to obtain light-trapping coatings of nanofibers by the copolymerization of dopamine and pyrrole, which can be directly and rapidly synthesized on a polystyrene (PS) foam at room temperature (Figure 9). Due to its excellent wettability, the coating is water permeable and can be directly applied on top of the PS insulator without the need for an additional water transport layer.

#### Membranes

Janus structural membranes with hydrophilic and hydrophobic surfaces are key structures for highly efficient SSGs. Such Janus membranes can be easily produced by filtering or coating hydrophilic membranes with hydrophobic photothermal material particles. Over the past years, various types of SSG membranes have been studied [37,39,45,54,56–57]. These SSG membranes can be applied to, for example, seawater desalination and photocatalytic degradation by utilizing the photothermal and separation functions of the membranes.

**Figure 10:**
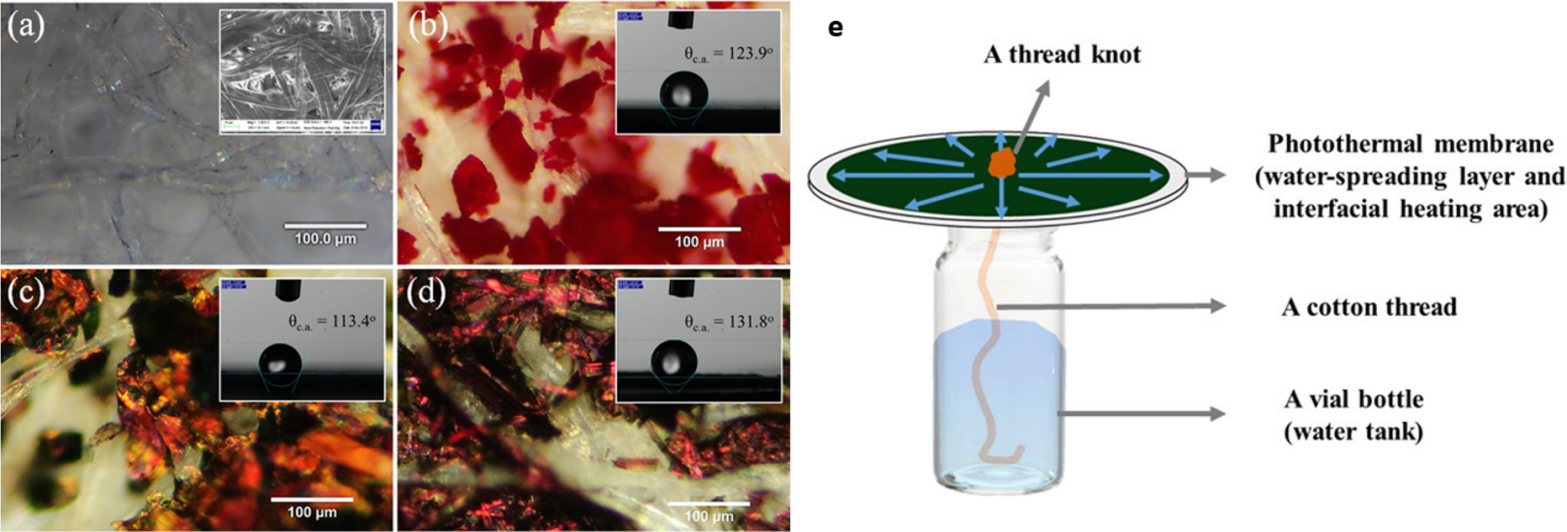
Surface morphology of (a) filter paper, (b) DPP-H, (c) DPP-DCV, and (d) DPP-INCN-based photothermal membranes visualized by optical microscopy. (e) Schematic illustration of the SSG device in this study. (Figure 10 was adapted with permission from [27], Copyright 2021 American Chemical Society).

As already described, DPP has been used as the PTM for SSG devices [27]. Microcrystalline solids of DPP molecules were formed in a solution and they were deposited on a filter paper by filtration. The filter papers with the deposited DPP derivatives were employed as photothermal films. Optical microscopy was employed to observe the surface structure of the films (Figure 10a–d). Figure 10a shows the filter paper. In the photothermal films, the filter paper is the supporting layer. The filter paper is indeed suitable for this purpose because the cellulose fibers are randomly oriented and many capillaries and pores can efficiently diffuse water. Note that the hydrophilic bottom part is employed for efficient water absorption and transport, and the hydrophobic top moiety is required for efficient steam generation. Since the DPP derivatives possess the siloxane chains, they are hydrophobic. The contact angle measurements revealed that the contact angles of the filter paper coated with DPP-H, DPP-DCV, and DPP-INCN were 123.9°, 113.4°, and 131.8°, respectively. In contrast, the contact angle of the bare filter paper remained at approx. 0°. These results suggest that coating the DPP derivatives on a filter paper produced Janus structures with a hydrophobic top surface suitable for vapor generation and a hydrophilic bottom surface for water uptake and transport. Figure 10e displays the developed SSG device. A cotton fiber thread connects the photothermal membrane to the water tank and supplies water to this photothermal film. The film is slightly lifted in order to reduce heat dissipation from the bulk water. This is also beneficial for SSG through the thermal energy generated by the PTM.

#### Porous 3D structures

Porous materials, such as sponges [45,49,58–59], foams [31,34,38,49–50,52–63], and aerogels [64–73], are also used as substrates for SSG devices. As the hydrophilic surface of porous structures is the key to efficient water transport through capillary forces, it is reasonable to combine them with conventional floating membranes. This is because PTMs are in most cases in contact with the bulk water. Moreover, 3D photothermal evaporators have superior properties to their 2D counterparts because solar steam evaporation can be facilitated by the increased surface area. Note that 3D evaporators enable efficient energy harvesting from the environment, and this is beneficial for water evaporation. Furthermore, when light is vertically irradiated, 3D evaporators provide an opportunity of water evaporation from the sidewalls that are not directly irradiated by light. The evaporation from the sidewalls leads to a lowering of the surface temperature of the sidewalls, allowing for further energy harvesting from the environment. All these processes will contribute to the increase in the overall water evaporation amount.

**Figure 11:**
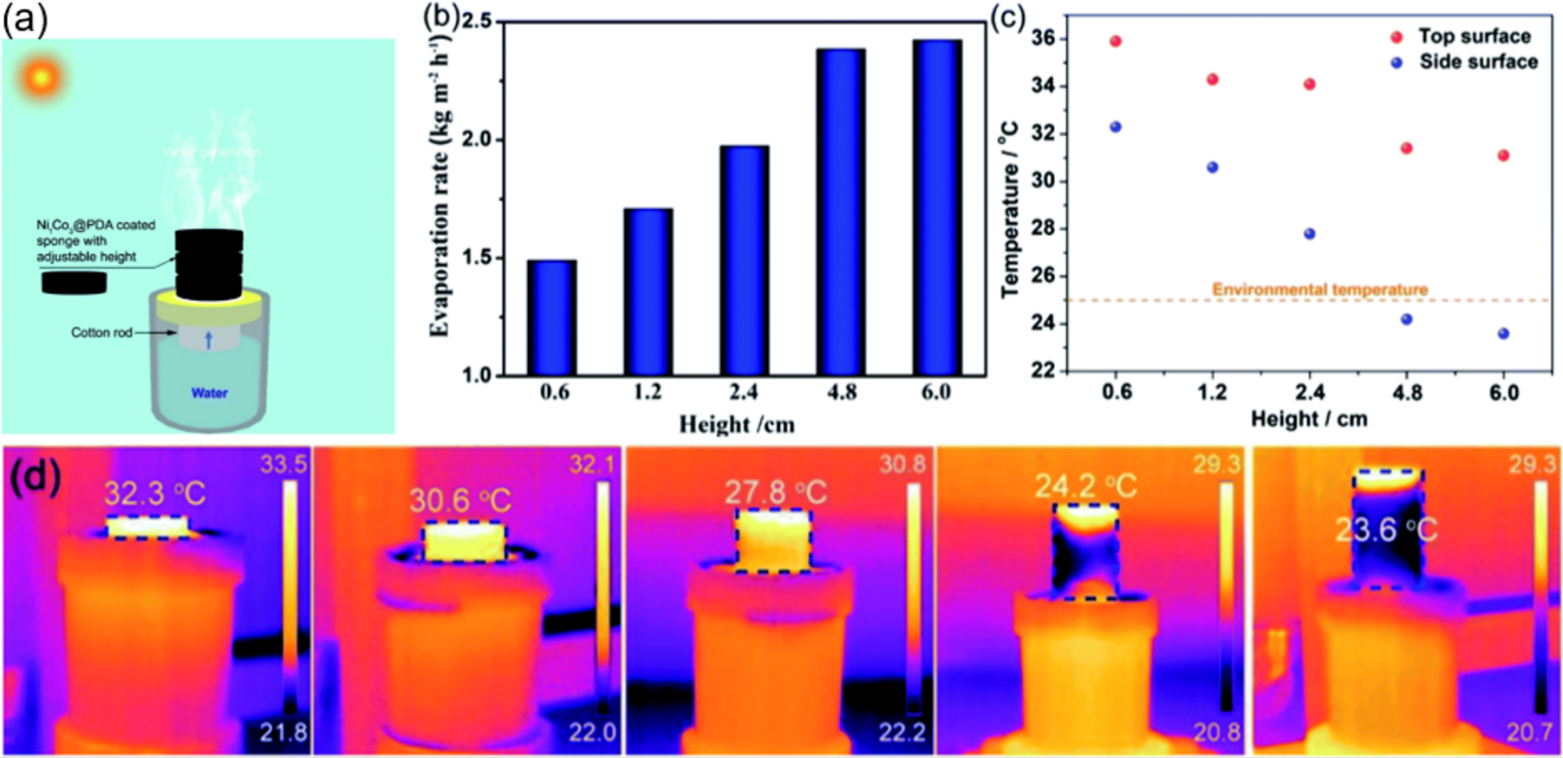
(a) Schematic illustration of a Ni_1_Co_3_@PDA-based photothermal sponge as light absorber. (b) Evaporation rates depending on the sponge thickness. (c) Average temperatures of the top and sidewall surfaces of the photothermal sponges with different heights under solar spectral irradiance (AM1.5G). (d) IR images of the photothermal sponges with the average temperatures of the sidewall surfaces under solar spectral irradiance (AM1.5G). Figure 11 was adapted with permission of The Royal Society of Chemistry from [49] (“Stackable nickel–cobalt@polydopamine nanosheet based photothermal sponges for highly efficient solar steam generation” by B. Shao et al., J. Mater. Chem. A, vol. 8, issue 23, © 2020); permission conveyed through Copyright Clearance Center, Inc. This content is not subject to CC BY 4.0.

Shao et al. fabricated a nickel–cobalt bimetal (Ni_1_Co_3_@PDA) coated with self-polymerized PDA as a PTM to improve the chemical stability and light absorption ability [49]. The Ni_1_Co_3_@PDA was dispersed onto the surface of a round commercial sponge by drop casting. The photothermal performance of the Ni_1_Co_3_@PDA deposited on the sponge was then evaluated using a kerosene lamp-like setup (Figure 11a). In this setup, a cotton rod was employed to connect between the sponge and bulk water. Thorough capillary forces, water is transported to the photothermal sponge. This is a simple design but efficiently minimizes the transport energy loss. In order to optimize the water evaporation rate and energy efficiency, the thickness of the photothermal sponge was changed in the range from 0.6 to 6.0 cm. It was then found that the evaporation rate increased from 1.49 to 2.42 kg·m^−2^·h^−1^ (Figure 11b). This was most likely because the average temperature of the sponge surface was lowered by the increased surface area of the sidewalls, which resulted in a reduced heat loss (Figure 11c and Figure 11d).

**Figure 12:**
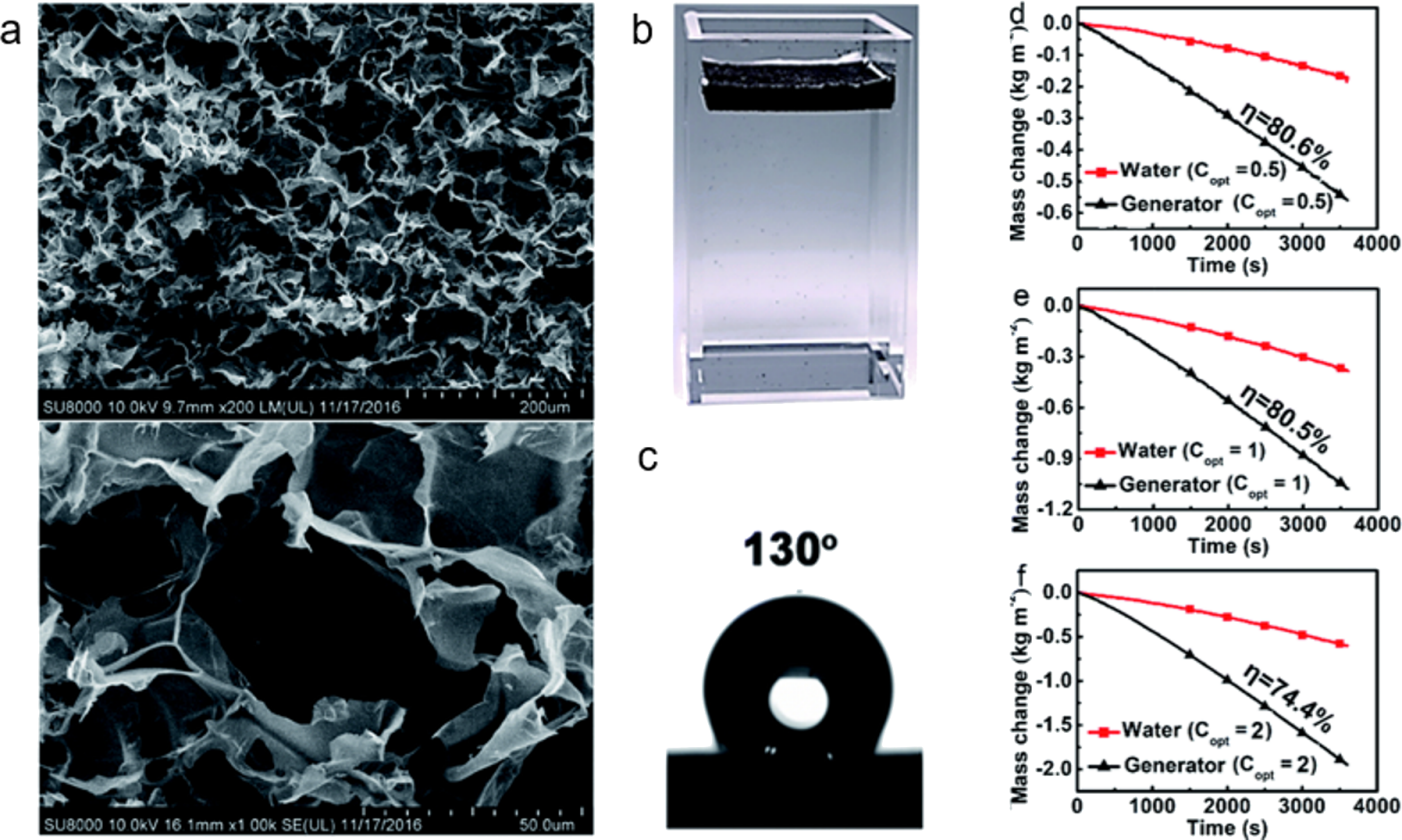
(a) SEM images of the poly(1,3,5-hexahydro-1,3,5-triazine)-based foams at low (up) and high (down) magnifications. (b) An optical image of the foam floating at the air–water interface. (c) The water contact angle of the foam. (d–f) Time-dependent mass changes with and without the evaporator foam under different optical concentrations. Figure 12 was adapted from [34] (“A durable monolithic polymer foam for efficient solar steam generation“, © The Royal Society of Chemistry 2018, by Q. Chen et al., distributed under the terms of the Creative Commons Attribution NonCommercial 3.0 Unported License, https://creativecommons.org/licenses/by-nc/3.0). This content is not subject to CC BY 4.0.

In 2018, Chen et al. used amine-terminated oligoanilines to obtain a type of poly(1,3,5-hexahydro-1,3,5-triazine) mainly consisting of an aniline trimer [34]. Undoped oligoanilines are hydrophobic, and this hydrophobicity allows the material to float on water. Microporous structures were obtained by NaCl particulate leaching. The pore sizes could be flexibly controlled by the size of the NaCl template. This method is simple, low-cost, and easy to scale up. The surface structures of the foam were visualized by SEM observations (Figure 12a). These materials can stably float at the air–water interface (Figure 12b). The water contact angle of the bulk materials was as high as 130° without any surface modification (Figure 12c). Hydrophobic foams are considered to have advantages over hydrophilic foams, such as a greater buoyancy due to their superior water resistance and antifouling properties. Due to the microporous nature of the polymer foams, after 10 min of swelling in water, the water could eventually be transported from the bottom to the surface through the foam’s interconnected pathways. These polymer evaporators achieved a high evaporation efficiency even at low solar intensities. Water could be continuously fed to the high-temperature section, and the evaporation rate remained constant after an initial transition period. At all light intensities, evaporation is greater with the evaporator than without the evaporator (Figure 12d–f).

## Conclusion

In this review, we presented the recent progress and important contributions regarding conjugated photothermal materials used in the SSG process, such as pyrrole derivatives, PANI, PPy, and PDA. Conjugated molecules can be designed to tailor the optical properties and hydrophilic/hydrophobic properties. Furthermore, conjugated hybrid materials exhibit excellent performance because of the synergistic effects of different components of the hybrid materials. The overall structural design concept of the absorbers was also described. The Janus structure reduces the heat loss to the bulk water, and the 3D design minimizes convection and radiation heat loss to the air, while at the same time gaining energy from the environment at the sidewalls.

As already described, high photothermal conversion efficiencies of the conjugated SSG absorbers have been reported. However, it is possible to further improve the photothermal conversion efficiency of conjugated molecules by systematically studying structure–property relationships. Optimized structures will be value-added high-performance photothermal materials. Also, they need to be hybridized with mass-produced active matrix materials, because low cost, reusability, chemical stability, and mass production will be required in the future. Furthermore, by designing the evaporator, it can be applied to various fields, such as seawater desalination, sterilization, and power generation. There is still room for research on how to optimize and extend this functionality. Combining solar energy and water, the most abundant resources on earth, SSG is expected to lead to a sustainable future through synergy with other solar devices and water treatment technologies.
